# Clinical risk stratification of paediatric renal transplant recipients using C1q and C3d fixing of de novo donor-specific antibodies

**DOI:** 10.1007/s00467-017-3772-7

**Published:** 2017-09-16

**Authors:** Jon Jin Kim, Olivia Shaw, Chloe Martin, George Michaelides, Ramnath Balasubramaniam, Neil J. Sebire, Nizam Mamode, Anthony Dorling, Robert Vaughan, Stephen D. Marks

**Affiliations:** 1 Department of Paediatric Nephrology, Nottingham University Hospital, Nottingham, UK; 2 Department of Paediatric Nephrology, Great Ormond Street Hospital for Children NHS Foundation Trust, WC1N 3JH, London, UK; 3 MRC Centre for Transplantation, Guy’s Hospital, London, UK; 4 Viapath Clinical Transplantation Laboratory, Guy’s Hospital, London, UK; 5 Department of Organisational Psychology, Birkbeck, University of London, London, UK; 6 University College London Great Ormond Street Institute of Child Health, London, UK

**Keywords:** Renal transplant, HLA antibodies, Donor-specific antibodies, Complement fixation, Prognosis

## Abstract

**Introduction:**

We have previously shown that children who developed de novo donor-specific human leukocyte antigen (HLA) antibodies (DSA) had greater decline in allograft function. We hypothesised that patients with complement-activating DSA would have poorer renal allograft outcomes.

**Methods:**

A total of 75 children developed DSA in the original study. The first positive DSA sample was subsequently tested for C1q and C3d fixing. The primary event was defined as 50% reduction from baseline estimated glomerular filtration rate and was analysed using the Kaplan–Meier estimator.

**Results:**

Of 65 patients tested, 32 (49%) and 23 (35%) tested positive for C1q and C3d fixing, respectively. Of the 32 C1q-positive (c1q+) patients, 13 (41%) did not show concomitant C3d fixing. The mean fluorescence intensity values of the original immunoglobulin G DSA correlated poorly with complement-fixing positivity (C1q: adjusted *R* ^2^ 0.072; C3d: adjusted *R* ^2^ 0.11; *p* < 0.05). C1q+ antibodies were associated with acute tubulitis [0.75 ± 0.18 (C1q+) vs. 0.25 ± 0.08 (C1q−) episodes per patient (mean ± standard error of the mean; *p* < 0.05] but not with worse long-term renal allograft dysfunction (median time to primary event 5.9 (C1q+) vs. 6.4 (C1q−) years; hazard ratio (HR) 0.74; 95% confidence ratio (CI) 0.30–1.81; *p* = 0.58]. C3d-positive (C3d+) antibodies were associated with positive C4d histological staining [47% (C3d+) vs. 20% (C3d−); *p* = 0.04] and with significantly worse long-term allograft dysfunction [median time to primary event: 5.6 (C3d+) vs. 6.5 (C3d−) years; HR 0.38; 95% CI 0.15–0.97; *p* = 0.04].

**Conclusion:**

Assessment of C3d fixing as part of prospective HLA monitoring can potentially aid stratification of patients at the highest risk of long-term renal allograft dysfunction.

**Electronic supplementary material:**

The online version of this article (10.1007/s00467-017-3772-7) contains supplementary material, which is available to authorized users.

## Introduction

Children represent a group of patients with low levels of sensitisation against human leukocyte antigens (HLA) as they have generally not been previously exposed to multiple blood products, pregnancies or previous transplants [1]. However, they do have a more naive immune compartment and are prone to developing infections which carry a small risk of cross-reaction with the allograft through heterologous immunity [2]. Children face lifelong immunosuppression and potential multiple re-transplants. Therefore, finding the correct balance between the suppression of alloimmunity and the side effects of immunosuppression is even more important [3].

We previously published the largest cohort of paediatric renal transplant recipients screened prospectively for de novo donor-specific HLA antibodies (DSA) [4]. DSA-positive patients were found to have a faster decline in allograft function and more features of antibody-mediated rejection (AMR) on biopsies done ‘for-cause’. Also, the level of allograft dysfunction correlated with rising mean fluorescence intensity (MFI) levels for Class II DSA. In the study reported here, we investigated further the capability of DSA to activate the complement cascade through in vitro assays detecting complement binding at the levels of C1q (first subcomponent of the C1 complex of the classical pathway of complement activation) and C3d (subcomponent of complement component 3) [5, 6]. We hypothesised that patients with complement-activating DSA would have poorer renal allograft outcomes.

## Materials and methods

### Study design

Patients who tested positive for DSA (DSA+) were identified from our previously published single-centre cohort study [4]. In brief, all renal transplant recipients from 1 January 2006 (existing and new transplants after this date) were screened prospectively (1–3, 6, 12 months post-transplant and annually thereafter) using OneLambda assays (One Lambda, Canoga Park, CA) and pan-immunoglobulin G (IgG) secondary antibody. The cumulative frequency of the antibody tests was 60, 86 and 98% at 3, 6 and 12 months, respectively. All sera were heat inactivated in a water bath at 56 °C for 30 min to alleviate prozone effects. No threshold MFI for positive DSA was set as an a priori criteria.

**Fig. 1:**
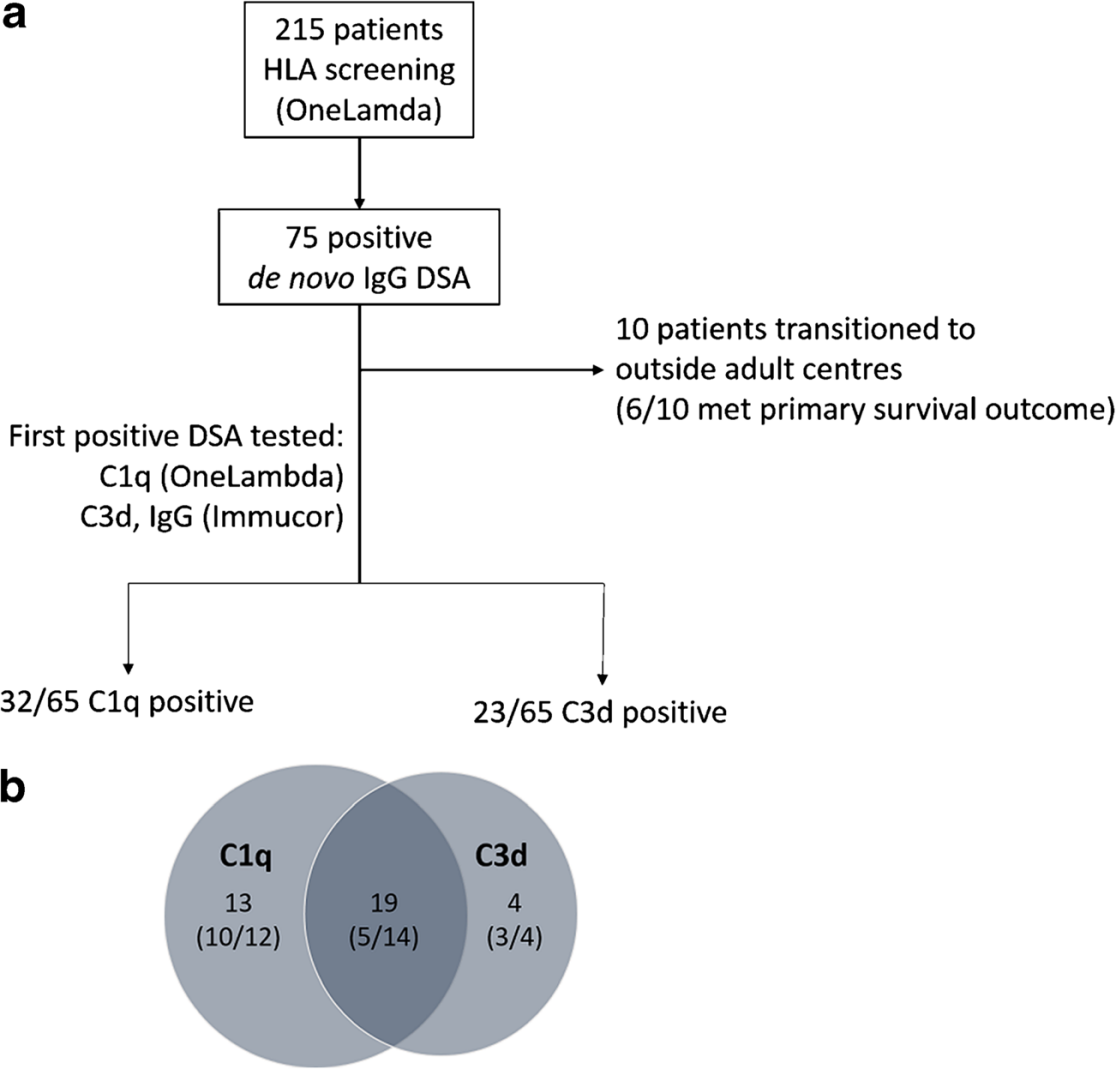
**a** Graphical representation of study design. **b** Venn diagram showing overlap between C1q (first subcomponent of the C1 complex of the classical pathway of complement activation) and C3d (subcomponent of complement component 3 C3) assay. *Numbers* indicate the number of Class I/Class II patients. *HLA* human leukocyte antigen, *IgG* immunoglobulin G, *DSA* donor-specific antibodies to human leukocyte antigen (HLA)

In this study, the first DSA-positive serum was further tested for complement binding capabilities (Fig. 1a). Clinical characteristics were as previously described. Follow-up estimated glomerular filtration rate (eGFR, calculated using Schwartz formula) data were extended until April 2015. Immunosuppression data were obtained at the time of DSA detection. Histological classification was based on the Banff 2009 criteria.

### C1q and C3d detection assays

Experiments were performed by researchers blinded to patient information at the Clinical Transplantation Laboratory, Viapath, Guy’s Hospital, London, in a single run and using assays from the same batch. Patients were defined as ‘complement positive’ if at least one DSA showed complement fixing.

C1q-binding DSA were identified using C1qScreen™ (One Lambda) according to the manufacturer’s protocol [5]. Sera were pre-treated with heat inactivation of the complement system as part of the protocol. Analysis was performed using the HLA Fusion 2.0 software (One Lambda). Complement positivity was assigned at >1000 MFI based on the negative control sera and internal negative control beads.

C3d-binding DSA were identified using Lifecodes C3d and Single Antigen assay (Immucor, London, UK) according to the manufacturer’s protocol [6]. In addition, sera were tested for pan-IgG using Lifecodes Single Antigen kits (Immucor). Results were analysed using the same manufacturer’s MatchIt software, and complement positivity was defined as per the software algorithm.

### Statistical analysis

Data were presented as the mean ± standard error of the mean) and as the median with the interquartile range (IQR), as appropriate. Comparisons between groups were performed with the Mann–Whitney test, and comparisons of proportions were performed using the Fischer and chi-square tests. Correlation between IgG MFI and complement positivity was estimated using logistic regression. Event-free survival was estimated with the Kaplan–Meier method and was compared between risk groups using the log-rank test. The primary event was defined as a sustained 50% reduction (defined as two consecutive results at least 3 months apart) from baseline eGFR as per the previous study results, and patients were censored at the end of the follow-up period [3]. Statistical analysis was performed using GraphPad Prism 5.0 (GraphPad Software Inc., LaJolla, CA), with *p* values of <0.05 considered to be significant. Multilevel linear modelling was performed using ‘nlme’ on the R statistical platform [7]. The model used individual patient nesting and fixed effect of follow-up time as described in our previous study [4]. The associations investigated were IgG MFI α C3d MFI and eGFR α C3d MFI over time.

## Results

### Complement binding results

**Fig. 2:**
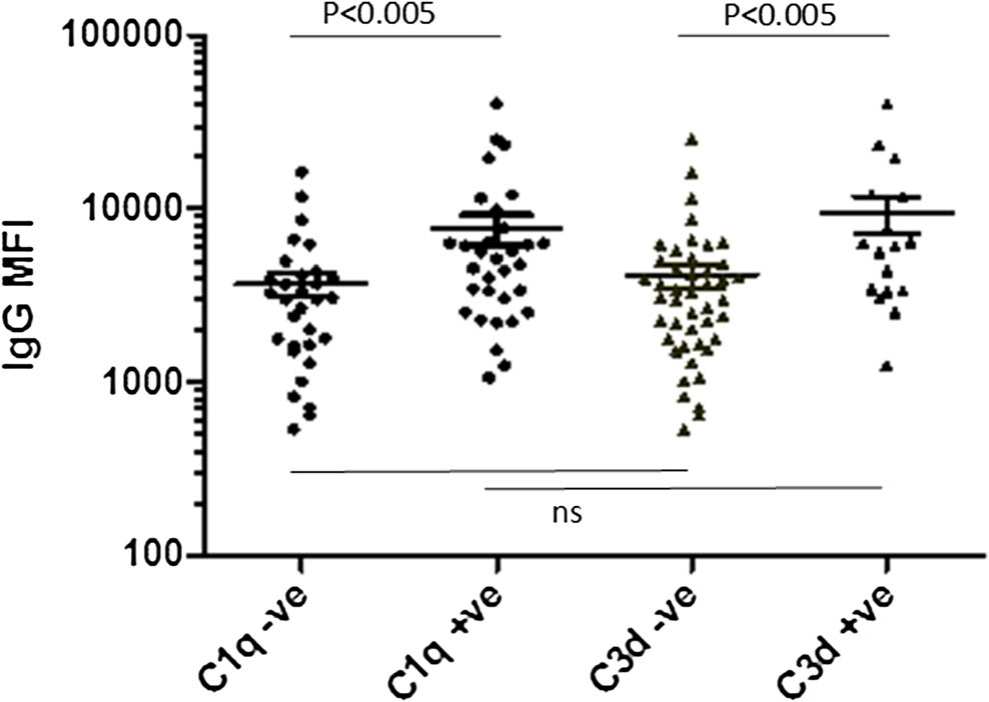
Corresponding IgG mean fluorescence intensity (*MFI*) values according to the complement binding results. Horizontal bars represents groups compared. *ns* Not significant

In our original cohort, 215 patients underwent prospective screening for HLA antibodies (at 1–3, 6, 12 months and annually thereafter), using the LABScreen Mixed screening tool followed by screening with the Single Antigen Beads (SAB) assay from OneLambda. Of these 215 patients, 75 tested positive for IgG DSA at a median time of 0.25 years post-transplant. Serum samples for 65 of these 75 patients were available for further testing, and the first positive DSA sample was tested using the C1q and C3d assays (Fig. 1a); serum samples for the remaining ten patients were unavailable due to these patients, and their sera, transferring to adult centres out of the region. This latter group of ten patients represented an older group (median age of transplant 13.7 years) with more cellular rejection [Electronic Supplementary Material (ESM) Table 1], of whom six met the primary outcome of 50% reduction from baseline eGFR. Using the C1q assay, 32 of the 65 (49%) patients included in the study tested positive for the following DSA: HLA-A (*n* = 5), HLA-B (*n* = 8), HLA-C (*n* = 2), HLA-DQ (*n* = 22) and HLA-DR (*n* = 4). Using the C3d assay, 23 of the 65 (35%) patients tested positive for the following DSA: HLA-A (*n* = 3), HLA-B (*n* = 5), HLA-DQ (*n* = 14) and HLA-DR (*n* = 4). The breakdown of HLA types according to the C1q and C3d assays was similar to that according to the whole DSA+ cohort assay (Table 1). Serum samples tested later post-transplant showed a tendency towards positive complement (C1q+/C3d+) results, although this trend was not statistically significant [Table 2 (median): 2.6 (C1q+) vs. 0.4 (C1q−) years, *p* = 0.17; 2.3 (C3d+) vs. 0.4 (C3d−) years, *p* = 0.28]. Higher total IgG MFI was observed for patients who had C1q+ DSA compared to those who had C1q− (mean ± SEM 4968 ± 1492 vs. 3006 ± 607, *p* < 0.005; Fig. 2) based on the original pan-IgG antibody identification. Nonetheless, there was a large overlap between MFI values and a poor correlation between IgG MFI and C1q results (adjusted *R* ^2^ = 0.072). Similarly, higher total IgG MFI was observed for patients with C3d+ DSA than for those with C3d− DSA (9483 ± 2289 vs. 4184 ± 648, *p* < 0.005) on the original OneLambda pan-IgG antibody identification; however, there was a poor correlation between IgG MFI and the C3d results (adjusted *R* ^2^ = 0.11). There was no difference between total IgG MFI in C1q− and C3d− patients, or in C1q+ and C3d+ patients. Therefore, it would appear that IgG MFI is not a significant predictor of DSA complement binding capabilities.

**Table 1:** Human leukocyte antigen types of C1q+ and C3d+ antibodies compared to the overall DSA+ cohort

HLA group	C1q+	C3d+	DSA+
HLA-A	5 (11%)	3 (12%)	16 (17%)
HLA-B	8 (20%)	5 (19%)	21 (22%)
HLA-C	2 (5%)	0	7 (8%)
HLA-DP	0	0	1 (1%)
HLA-DQ	22 (54%)	14 (54%)	34 (37%)
HLA-DR	4 (10%)	4 (15%)	14 (15%)

*p* = 0.5, Chi-square test

C1q, First subcomponent of the C1 complex of the classical pathway of complement activation; C3d, subcompent of complement component 3 C3; HLA, human leukocyte antigen; IgG, immunoglobulin G; DSA, donor-specific HLA antibodies

**Table 2:** Clinical characteristics of patients according to assay results for C1q and C3d

Clinical characteristics of patients	C1q		C3d	
	C1q+ (*n* = 32)	C1q− (*n* = 33)	C3d+ (*n* = 23)	C3d− (*n* = 42)
Time to first DSA (years)	2.6 (0.1–4.9)	0.4 (0.1–2.1)	2.3 (0.1–4.1)	0.4 (0.1–3.1)
Sex, male	21 (66%)**	27 (82%)**	15 (65%)	33 (79%)
Cause of end-stage kidney disease				
CAKUT	22 (69%)*	12 (36%)*	15 (65%)	19 (45%)
Glomerulonephritis	3 (9%)	5 (15%)	3 (13%)	5 (12%)
Others	7 (22%)	16 (48%)	5 (22%)	18 (43%)
Mismatches	2 (2–3)	2 (2–3)	2 (2–3)	2 (2–3)
Age of transplant (years)	7.2 (4.7–10.5)	11.1 (5.6–13.9)	7.3 (5.1–10.1)	10.3 (5.2–13.8)
Donor type LD	20 (63%)	17 (52%)	14 (61%)	23 (55%)
Medication:				
Pred/Aza/Tac	7 (24%)	11 (35%)	3 (18%)*	15 (35%)*
Pred/Tac/MMF	5 (17%)	4 (13%)	4 (24%)*	5 (12%)*
Pred/MMF	8 (28%)	5 (26%)	6 (35%)*	7 (16%)*
Pred/Tac	6 (21%)	10 (32%)	2 (12%)*	14 (33%)*
Tac/MMF	1 (3%)	1 (3%)	0	2 (5%)
Tac	1 (3%)		1 (5%)	
MMF	1 (3%)		1 (5%)	

*, ** Significantly different at: *p < 0.05, **p < 0.005. Results are not significantly different unless otherwise stated

Results in table are presented at the median with the interquartile range (IQR) in parenthesis or as the frequency (number) with the percentage in parenthesis, as appropriate

CAKUT, Congenital anomalies of the kidney and urinary tract; LD, living donor;; Pred, prednisolone; Aza, azathioprine; Tac, tacrolimus; MMF, mycophenolate mofetil

Comparison of the C1q and C3d results revealed that 19 patients were C1q+/C3d+, 13 patients were C1q+/C3d− and four patients were C3d+/C1q− (Fig. 1b). Therefore, a large proportion of patients with positive C1q binding did not show concomitant C3d binding (13/32, 41%). The breakdown of individual DSA classes are as shown in Table 1. There was a better concordance between C1q and C3d binding for Class II DSA. As the single antigen beads differ due the different manufacturers of the C1q and C3d assays, all sera were retested for DSA IgG binding using the SAB assay from the C3d manufacturer in order to confirm antibody detection and to rule out the detection of potential false positives due to the differing manufacturing methods between the kits. For C1q+/C3d− patients, 17/22 (77%) DSA IgG specificities were detected using the assays of both manufacturers. For C1q−/C3d+ patients, 3/7 (43%) DSA IgG specificities were detected using the assays of both manufacturers. Of note, there were no samples which were positive for complement binding and negative for DSA IgG detection, suggesting that no non-IgG DSA were detected.

### Clinical characteristics

The clinical characteristics of patients grouped according to complement binding are shown in Table 1. There were no differences in age at transplantation, donor type and number of mismatches. In terms of C1q, there were more boys and fewer congenital anomalies of the kidney and urinary tract (CAKUT) in the C1q− group than in the C1q+ group. In terms of immunosuppression at the time of DSA detection, roughly half of patients were on dual therapy consisting of prednisolone and either tacrolimus or mycophenolate mofetil (MMF). Patients were switched to MMF to minimise calcineurin inhibitor toxicity, which is in line with standard clinical practice at that time [8, 9]. Taking into consideration the small numbers in the different groups, the differences in medications were statistically significant in the patients tested for C3d. C3d+ patients were more likely to be on MMF than on tacrolimus when on dual therapy [6/8 (75%) C3d+ vs. 7/21 (33%) C3d−; *p* < 0.05]. The proportion of patients on azathioprine was higher in the C3d− group than in the C3d+ group, compared to MMF for patients on triple therapy [3/7 (48%) C3d+ vs. 15/20 (75%) C3d−; *p* < 0.05].

### Clinical outcomes

**Fig. 3:**
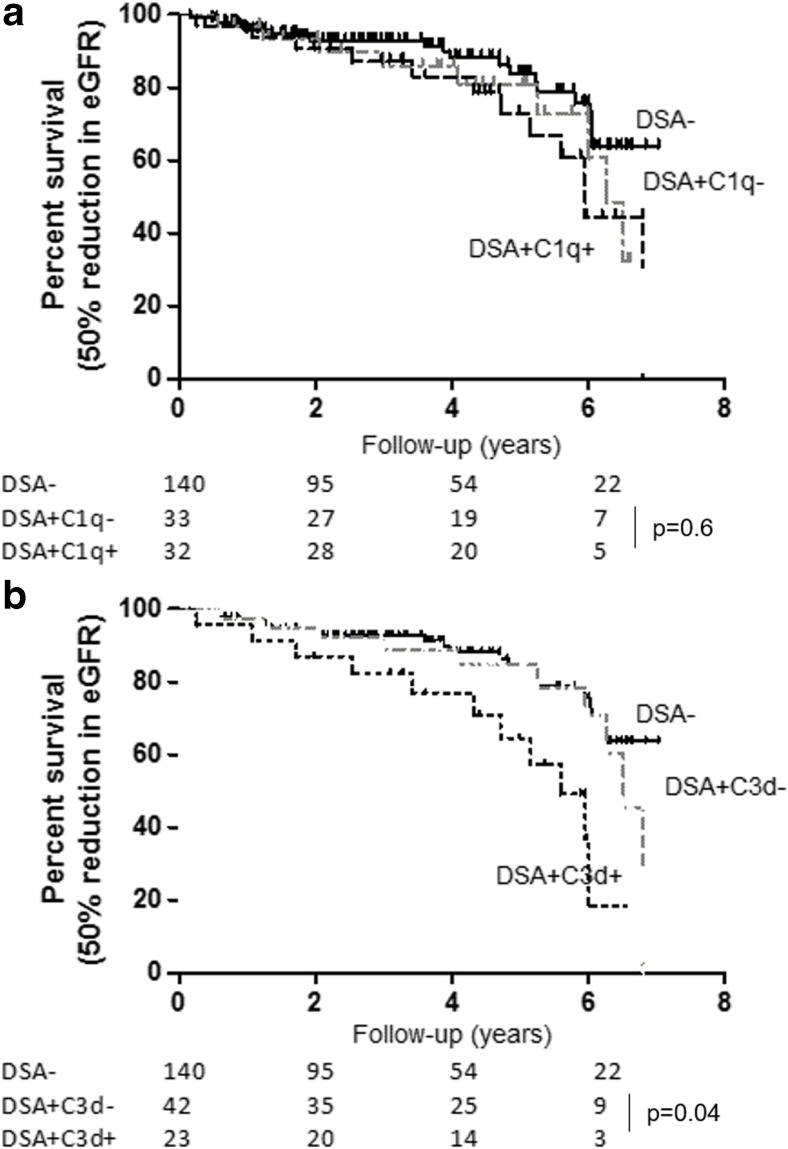
Time to event (defined as a 50% reduction from baseline estimated glomerular filtration rate (*eGFR*) according to DSA−, DSA+/C1q− and DSA+/C1q+ (**a**) and DSA−, DSA+/C3d− and DSA+/C3d− (**b**)

The primary outcome was a 50% reduction in eGFR, which was used as a surrogate marker of long-term renal allograft survival (Fig. 3). Patients who were DSA− from the previous study were used in the present study for comparative purposes. There was no difference in eGFR decline between C1q+ and C1q− patients [median time to primary event 5.9 vs. 6.4 years, respectively; *p* = 0.58; hazards ratio (HR) 0.74; 95% confidence interval (CI) 0.30–1.81]. On the other hand, C3d+ patients had a significantly faster eGFR decline than C3d− patients (5.6 vs. 6.5 years, *p* = 0.04; HR 0.38; 95% CI 0.15–0.97). Combining the C1q and C3d results did not improve the significance of the complement binding assays (C1q+/C3d+ vs. C1q−/C3d−: 5.2 vs. 6.6 years respectively, *p* = 0.09; HR 0.42; 95% CI 0.15–1.14) (ESM Fig. 1). Counter-intuitively, single binding of only either C1q or C3d (C1q+/C3d− and C1q−/C3d+) did not adversely affect renal allograft function.

Histology findings based on complement binding results are shown in Table 3. C1q+ patients were associated with increased episodes of tubulitis [0.75 ± 0.18 (C1q+) vs. 0.25 ± 0.08 (C1q−) episodes per patient; *p* < 0.05]. Patients who were positive for complement binding showed a higher proportion of C4d binding on biopsies, which reached statistical significance for the C3d results [48% (C3d+) vs. 20% (C3d−); *p* < 0.05). There was no difference in AMR (composite of glomerulitis, pericapillaritis and glomerular double contouring) or presence of CD20 aggregates.

**Table 3:** Histological findings based on complement binding results

Histological findings	C1q		C3d	
	C1q+ (*n* = 32)	C1q− (*n* = 33)	C3d+ (*n* = 23)	C3d− (*n* = 42)
Tubulitis	0.75 ± 0.18*	0.25 ± 0.08*	0.65 ± 0.22	0.42 ± 0.22
Vasculitis	0*	0.18 ± 0.09*	0.1 ± 0.07	0.16 ± 0.07
C4d	13 (39%)	6 (19%)	10 (48%)*	9 (20%)*
AMR	8 (24%)	3 (9%)	5 (24%)	6 (14%)
CD20	10 (30%)	9 (28%)	7 (33%)	12 (27%)

*Significantly different at *p* < 0.05. Results are not significantly different unless otherwise stated

Results in the table are shown as the mean number of episodes per patient ± standard error of the mean or as a number (frequency) with the percentage in parenthesis

AMR, Antibody-mediated rejection

### Longitudinal analysis

Based on the better correlation with outcome obtained with the C3d assay, testing for C3d was extended to all DSA+ sera. Of the 65 patients enrolled in the study, 33 had multiple DSA+ sera available for testing (median 3, IQR 3–4 sera per patient). Ten patients remained C3d−; four patients converted from negative to positive; 12 patients were C3d+ throughout; four patients were intermittently positive; three patients were positive at the start, then became negative. The latter three patients had low C3d MFI of 1979 (HLA-B), 1750 (HLA-DR) and 1386 (HLA-DQ). Four patients received intravenous rituximab—three for chronic AMR and one for post-transplant lymphoproliferative disorder. C3d remained positive in three patients who received intravenous rituximab; the remaining patient had low C3d+ DSA (MFI 1386) which became negative after receiving an intravenous infusion of rituximab and increasing immunosuppression to prednisolone, tacrolimus and MMF. Over time, the increase in C3d MFI correlated with increasing IgG MFI (co-efficient 1.5, ±1.1 units; *p* < 0.0001). There was no correlation between eGFR and C3d MFI (1.0 ± 1.5 ml/min/1.73 m^2^; *p* = 0.9).

## Discussion

We investigated the utility of complement binding assays to further stratify DSA+ patients at risk of worse renal allograft outcomes. In vitro, a larger proportion of DSA fixed C1q compared to C3d (49 vs. 35%). Complement positivity correlated poorly with IgG MFI, and a clear threshold could not be defined. C1q+ DSA were associated with a higher risk of tubulitis, but the long-term renal allograft function of these patients was not significantly different to those with C1q− DSA. C3d+ DSA were associated with more C4d staining on ‘for-cause’ biopsies and significantly worse renal allograft function.

We hypothesised that patients producing complement-fixing DSA would have a worse allograft outcome because of the implication that in vivo, binding to the allograft endothelium would enable recruitment of additional immune activation pathways through activation of the complement cascade. The ability of antibody to activate the complement cascade depends on the IgG subtype and corresponding Fc portion [10], in addition to the target antigen density and proximity of multiple target HLA molecules to allow cross-linking and subsequent binding of C1q [10]. This in turn triggers the classical complement pathway through a series of regulated intermediate steps, leading to the formation of C4bC2b, which catalyses the breakdown of C3 into its active components [11]. C3 is the converging point of three complement pathways, ultimately leading to the generation of C5b-9, the membrane attack complex (MAC) and cell lysis. The breakdown products of C4b and C3 include C4d and C3d, respectively, which covalently bind to cell membranes [11]. Therefore, in vitro tests have been designed to probe the complement binding capabilities of DSA at the check-points C1q, C4d and C3d [12]. Fixing of C1q is a pre-requisite for initiation of the complement cascade. C3d itself is dissociated from IgG, i.e. C3d is not bound to antibody but rather is attached to cell membranes or complement receptors. Interestingly, we showed that 41% of antibodies positive for C1q binding do not concomitantly fix C3d, despite agreement between the pan-IgG kits. Whether this is a specific difference in functional characteristics of the antibody detected, a reflection of antibody titre or a difference inherent in the assays needs addressing in the future.

This study focused on DSA+ patients initially identified using the pan-IgG LABScreen SAB. We therefore cannot rule out patients who might have tested DSA IgG+ using the alternative Immucor SAB assay. However, our laboratory experience and a review of the literature suggests agreement rates of 90% [13]. In our subset of C1q−/C3d+ patients, there was only 50% agreement between kits, i.e. additional IgG+ patients were identified on the Immucor SAB. However, long-term renal allograft function was not worse. Therefore, the discrepancy in results could be due to kit-related factors, such as the presence of denatured HLA antigen or the detection of potentially non-significant antibody [14]. In addition, complement-binding DSA tended to be detected later post-transplant, in agreement with previous studies showing that patients with early onset DSA and AMR had better responses to treatment and favourable clinical outcomes [15–17].

This study is limited by its retrospective nature and by the relatively small number of patients, which precluded further multi-variable survival analysis. In addition, rejection and reduced allograft function was more prevalent among the group of patients for whom sera were unavailable for testing, whom we hypothesised would have complement-binding DSA. With these caveats, our data do suggest an association between C1q+ DSA and increased episodes of acute tubulitis. This was also reported by Lefaucheur et al. using a non-supervised principal component analysis examining IgG subclasses and C1q [18]. C4d staining in vivo correlated with C3d testing in vitro which is downstream of C4 in the complement cascade. In vivo C3d staining has rarely been reported in the literature. One histological study of biopsies during acute allograft rejection showed C3d peritubular capillary staining in 30% of samples and increased renal allograft loss in the C3d+ patients [19]. MAC deposition has been shown in patients with HLA incompatible transplants, but its role in non-sensitised de novo AMR has not been studied [20]. MAC deposition results in cell lysis, although sublytic concentrations also can induce pro-inflammatory changes in glomerular cells and endothelial mesenchymal transition of tubular cells [21, 22].

The results of our study are in concordance with those of a recently published study in paediatric renal transplant recipients that also concomitantly assessed C1q and C3d binding [23]. Both studies showed a lower proportion of C3d binding and better prognostic predictability with the C3d assay. Comoli et al. also showed that patients could progress from C1q−/C3d− to C1q+/C3d+ and from C1q+/C3d− to C1q+/C3d+. Some patients were intermittently positive for complement binding associated with a low MFI of <2000 [23]. The current studies highlight the complexities of assessing DSA. MFI has often been used in studies as a quantitative measure of DSA although there is significant inter-assay variability and the assay is not licenced clinically as a quantitative measure [13]. It is also subject to the prozone effect which can give an artificially low MFI [24]. This can be overcome by dilutional titering. although this adds additional time and cost factors. In addition, studies have shown an association between C1q binding, IgG subtypes and IgG MFI, thus limiting the extra information obtainable in performing all three assays [25]. In our study, sera were obtained prospectively as per guidelines regardless of renal allograft function; as compared to studies which were done at the time of graft dysfunction and biopsy ‘for cause’ [6, 26]. In addition, our patients had low pre-transplant HLA antibody sensitisation rates which are not comparable to those of adult studies which include highly-sensitised and HLA-incompatible transplants [4, 5, 17]. We showed that the C3d assay potentially further stratified patients at the highest risk of renal allograft failure. This is independent of IgG MFI as the correlation between C3d and IgG MFI was poor (adjusted *R* ^2^ 0.11), with a significant overlap of C3d+ and C3d– patients in the moderate MFI range of between 1000 and 8000. Nonetheless, the results would be strengthened by being validated in a prospective study.

In conclusion, our study adds to the evidence of the potential importance of determining complement binding capabilities when testing for de novo DSA. Of the DSA we detected 49% could bind C1q, and its presence was associated with an increased proportion of ‘for-cause’ biopsies showing acute tubulitis, but not with worse long-term outcome. 35% of the DSA fixed C3d, and these DSA were associated with an increased proportion of ‘for-cause’ biopsies demonstrating positive C4d histological staining and significantly worse long-term renal allograft dysfunction. With the increasing financial pressures on healthcare provision, along with the significant costs of performing these tests, we believe these data may aid the decision-making behind the choice of tests used for post-transplant DSA monitoring.

## Electronic supplementary material

Supplementary Table 1 Clinical characteristics of the 10 patients who were not included in this study due to their sera being not available for complement testing (DOCX 12 kb)

Supplementary Figure 1 Time to event (defined as a 50% reduction from baseline eGFR) according to combined C1q and C3d results (GIF 26 kb)

High resolution image (TIFF 491 kb)
