# Supplementary material for: Clinical risk stratification of paediatric renal transplant recipients using C1q and C3d fixing of de novo donor-specific antibodies
Source: Pediatr Nephrol. 2017 Sep 16;33(1):167–74. doi: 10.1007/s00467-017-3772-7 (PMC5700253; doi:10.1007/s00467-017-3772-7)
Supplement: Supplementary file 1 — Clinical characteristics of the 10 patients who were not included in this study due to their sera being not available for complement testing (DOCX 12 kb) [file 467_2017_3772_MOESM1_ESM.docx]

|  | Patients with unavailable sera (n=10) |
| --- | --- |
| Sex M (%) | 4 (40%) |
| Cause of end-stage kidney disease  CAKUT  Glomerulonephritis  Others | 3 1 6 |
| Mismatches  Median (IQR) | 3 (2-3) |
| Age transplant  Median (IQR) | 13.7 (10.0 – 14.8) |
| Donor type LD (%) | 3 (30%) |
| Tubulitis (median, IQR number of episodes per patient) | 2 (1 – 3.5) |
| Vasculitis (median, IQR number of episodes per patient) | 1 (0 – 1.5) |
| C4d (%) | 2 (20%) |
| AMR (%) | 4 (40%) |
| CD20 (%) | 3 (30%) |

**Supplementary Table 1:** Clinical characteristics of the 10 patients who were not included in this study due to their sera being not available for complement testing
