# Supplementary figures and images for: Clinical risk stratification of paediatric renal transplant recipients using C1q and C3d fixing of de novo donor-specific antibodies
Source: Pediatr Nephrol. 2017 Sep 16;33(1):167–74. doi: 10.1007/s00467-017-3772-7 (PMC5700253; doi:10.1007/s00467-017-3772-7)

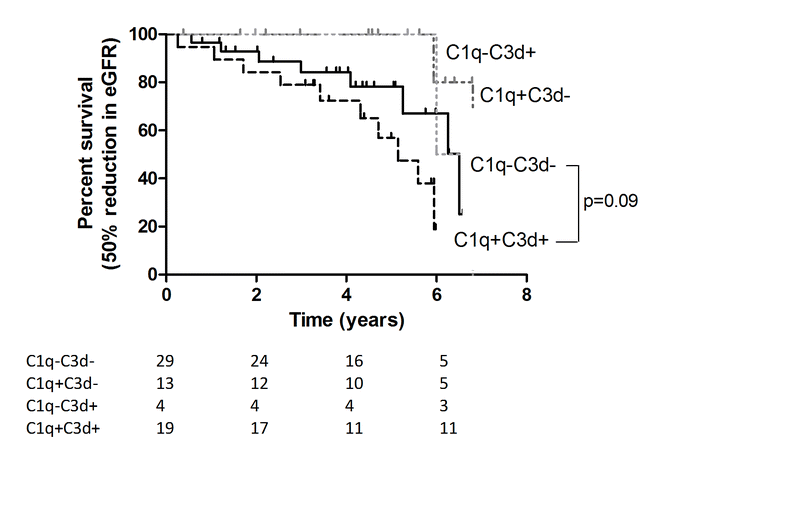

Supplement: Supplementary file 2 — Time to event (defined as a 50% reduction from baseline eGFR) according to combined C1q and C3d results (GIF 26 kb) [file 467_2017_3772_Fig4_ESM.gif]

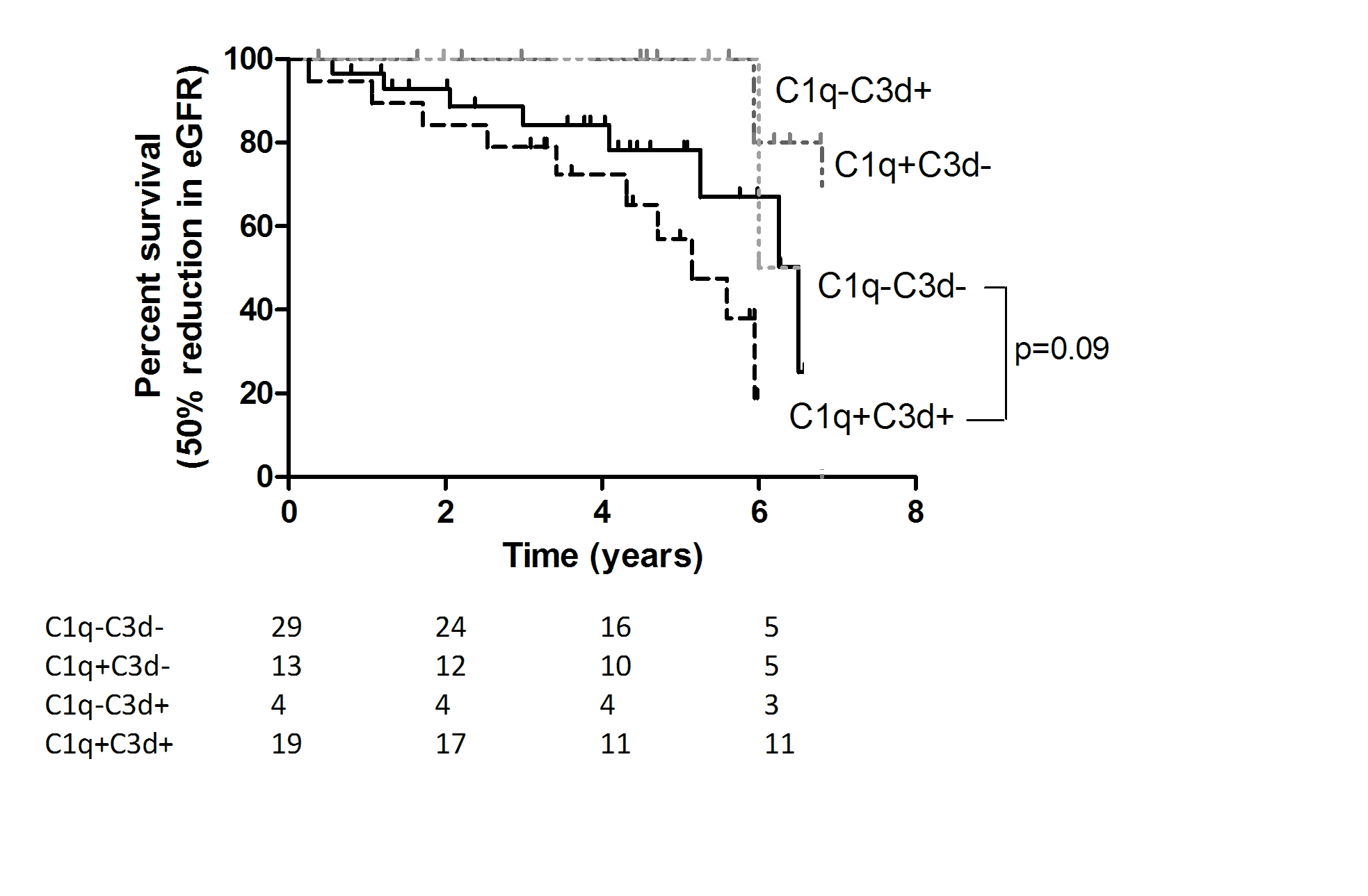

Supplement: Supplementary file 3 — High resolution image (TIFF 491 kb) [file 467_2017_3772_MOESM2_ESM.tif]
